# A 360-degree perspective on adeno-associated virus (AAV)-based gene therapy for haemophilia: Insights from the physician, the nurse and the patient

**DOI:** 10.1186/s13023-024-03181-2

**Published:** 2024-05-13

**Authors:** Wolfgang Miesbach, Greta Mulders, Daan Breederveld, Karen Pinachyan, Sandra Le Quellec, Ingrid Pabinger

**Affiliations:** 1 Coagulation and Haemophilia Centre, Medical Clinic 2, Goethe University Hospital, Frankfurt am Main, Germany; 2 Department of Hematology, Erasmus University Medical Center, Rotterdam, The Netherlands; 3 Brendaan Occupational Health Consultancy, Amsterdam, The Netherlands; 4 CSL Behring Europe, Hattersheim-am-Main, Germany; 5 Clinical Division of Haematology and Haemostaseology, Department of Medicine I, Medical University of Vienna, Vienna, Austria

**Keywords:** Adeno-associated virus, Gene therapy, Haemophilia, Multidisciplinary, Patient care

## Abstract

**Background:**

Adeno-associated virus (AAV)-based gene therapy for haemophilia has advanced substantially in the last 13 years; recently, three products have received approvals from regulatory authorities. Although the impact on quality of life seems promising, some limitations remain, such as the presence of pre-existing anti-AAV neutralising antibodies and the occurrence of hepatotoxicity. This review follows the CSL Behring-sponsored symposium at the 27th Congress of the European Hematology Association (EHA) 2022 that examined the haemophilia gene therapy process from a 360-degree multidisciplinary perspective. Here, the faculty (haematologist, nurse and haemophilia patient) summarised their own viewpoints from the symposium, with the aim of highlighting the key considerations required to engage with gene therapy effectively, for both patients and providers, as well as the importance of multidisciplinary collaboration, including with industry.

**Results:**

When considering these new therapies, patients face a complex decision-making process, which includes whether gene therapy is right for them at their current stage of life. The authors agreed that collaboration and tailored education across the multidisciplinary team (including patients and their carers/families), starting early in the process and continuing throughout the long-term follow-up period, is key for the success of gene therapy. Additionally, patient expectations, which may surround eligibility, follow-up requirements and treatment outcomes, should be continually explored. During these ongoing discussions, transparent communication of the unknown factors, such as anticipated clotting factor levels, long-term factor expression and safety, and psychological changes, is critical. To ensure efficiency and comprehensiveness, clearly‑defined protocols should outline the whole process, which should include the recording and management of long-term effects.

**Conclusion:**

In order to engage effectively, both patients and providers should be familiar with these key considerations prior to their involvement with the haemophilia gene therapy process. The future after the approval of haemophilia gene therapies remains to be seen and real-world evidence is eagerly awaited.

## Background

Haemophilia is caused by mutations in the genes encoding coagulation factor VIII (FVIII) or IX (FIX) [1]. The current standard of care for people with severe haemophilia is life-long prophylaxis with recombinant or plasma-derived coagulation factor concentrate, representing considerable treatment burden without eliminating the risk of bleeding [1, 2]. In addition, development of inhibitors to the administered coagulation factor concentrate may complicate treatment [3]. In the last 13 years, adeno-associated virus (AAV)-based gene therapy for haemophilia, which involves the transfer of genes for FVIII or FIX to target cells following a single infusion, has advanced substantially [2, 4]; the aim of which is to enable long-term endogenous coagulation factor production [4]. After haemophilia gene therapy, patients have reported improvements in their quality of life, bleeding rate and physical activity, as well as more freedom [5]. Although this suggests that gene therapy could have a positive impact on the lives of patients living with haemophilia, there are numerous other aspects that are essential to consider.

Positive outcomes have been reported in Phase 3 trials of three products. In 132 adult males with haemophilia A, the GENEr8-1 study showed that valoctocogene roxaparvovec increased FVIII activity from baseline (when participants were receiving FVIII prophylaxis) to Month 24 post-treatment by a mean of 22.0 IU/dL and 35.1 IU/dL using chromogenic and one-stage assays, respectively [6]. Additionally, mean annualised FVIII concentrate consumption decreased by 98.2% and mean annualised rate of treated bleeding episodes was reduced by 84.5% from baseline (*n* = 112). In the HOPE-B study, 54 adult males with haemophilia B were treated with etranacogene dezaparvovec following a ≥ 6-month lead-in period with FIX prophylaxis [7]. Annualised bleeding rate reduced from 4.19 during the lead-in period to 1.51 post-treatment (Months 7–18) and was sustained at Month 24; 96.3% of study participants stopped and remained free from prophylactic FIX therapy over 24 months post-treatment [7, 8]. At Months 6 and 24, mean FIX activity levels increased to 39.0 IU/dL and 36.7 IU/dL, respectively [7, 8]. Positive outcomes were also reported from the Phase 3 BENEGENE-2 trial investigating fidanacogene elaparvovec in adults with haemophilia B, with relatively stable FIX activity levels observed at Month 24 in the 22/45 patients for which data were reported; mean FIX activity level using the one-stage SynthASil assay was 25.0% in these patients [9].

In August 2022, valoctocogene roxaparvovec (ROCTAVIAN™; BioMarin Pharmaceutical Inc.) received conditional approval from the European Commission (EC) for use in adults with haemophilia A; approval by the US Food and Drug Administration (FDA) followed in June 2023 [10, 11]. In November 2022, etranacogene dezaparvovec (HEMGENIX^®^; CSL Behring) received FDA approval for use in adults with haemophilia B, before conditional approval by the EC and UK Medicines and Healthcare products Regulatory Agency (MHRA) in early 2023 [12–14]. In October 2023, Health Canada authorised etranacogene dezaparvovec [15], which was followed by authorisation by Swissmedic in January 2024 [16]. In December 2023, Health Canada approved fidanacogene elaparvovec (BEQVEZ™; Pfizer Canada ULC) for use in adults with haemophilia B [17].

Despite positive outcomes, challenges remain, and the pharmaceutical industry is focused on facilitating wider treatment access for haemophilia patients. Valoctocogene roxaparvovec, etranacogene dezaparvovec and fidanacogene elaparvovec are approved for use only in adults without factor inhibitors [10–13, 17]. Animal models suggest that AAV-based gene therapy for haemophilia A has the potential for induction of immune tolerance to FVIII [18]. Of note, a study evaluating valoctocogene roxaparvovec in patients with severe haemophilia A and FVIII inhibitors is in the recruitment stage (NCT04684940) [19].

Although it has been shown that pre-existing anti-AAV neutralising antibodies (NAbs) can impair therapeutically useful vector delivery [20], the presence of these antibodies (up to a titre of 678) did not affect treatment efficacy with etranacogene dezaparvovec [7]. Research is still ongoing within this area for valoctocogene roxaparvovec and currently this product is only indicated for use in patients without pre-existing anti-AAV5 NAbs [6, 10, 11]. Similarly, Health Canada has only approved the use of fidanacogene elaparvovec in patients without anti-AAV NAbs [17].

The extent of transgene expression can vary between patients [20]; monitoring of coagulation factor levels is needed post-treatment [10–13, 17]. The three licensed products have associated risks of liver toxicity, which may be accompanied by reduced expression of the transgenic protein; this requires post-infusion monitoring of liver enzymes and potentially corticosteroid treatment, introducing further risk of adverse events [10–13, 17, 20]. Cytotoxic T-cell responses against the AAV capsid is one suggested explanation for this hepatotoxicity [20]. Although it is thought that AAV vectors remain mostly episomal in the nucleus, vector integration can occur and it is unknown whether there is an increased risk of genotoxicity [2, 20]. Larger, long-term follow-up studies will be essential for gaining a more comprehensive understanding of the outcomes and safety of gene therapy [20, 21], and partnerships across industry, patient organisations and regulatory authorities will be crucial for their success [21].

It is important to note that gene therapy has not been studied in children with haemophilia [20], but paediatric investigation plans have been agreed by the European Medicines Agency [22–24]. The use of gene therapy in young people could avert long-term morbidity by preventing bleeds from an early age [20].

A further consideration is the cost of haemophilia gene therapy. Alternative payment models have been proposed due to the associated clinical and economical unknowns; introduction of these new methods will require engagement of all stakeholders [25]. One such model is outcome-based contracting, which involves the reimbursement of costs if expected outcomes are not met, thereby achieving value for patients [25–27].

Lastly, AAV-based gene therapy is irreversible and a one-time only treatment due to priming of the recipient’s immune system to the vector [21, 28]. Thus, patient expectations must be thoroughly explored in order to prevent “buyer’s remorse”, for example in the scenario of products becoming inferior to newly developed AAV vectors [21, 28].

During the CSL Behring-sponsored symposium at the 27th Congress of the European Hematology Association (EHA) 2022 in Vienna, Austria, Ingrid Pabinger (Symposium Chair, haematologist), Wolfgang Miesbach (haematologist), Greta Mulders (nurse) and Daan Breederveld (haemophilia patient and occupational health physician) described gene therapy from a 360-degree perspective of the haemophilia multidisciplinary team (MDT). A 360-degree perspective allows individuals involved in patient care to have oversight of the patient journey and of each MDT member’s role. In this review, the faculty summarised their own viewpoints from the symposium, focusing on the importance of the 360-degree perspective, the expectations and unknowns of gene therapy and the practical steps needed for gene therapy delivery. This review aims to highlight the key considerations required to engage with gene therapy effectively, for both patients and providers, as well as the importance of multidisciplinary collaboration, including with industry.

### Gene therapy in practice: The 360-degree perspective

The introduction of gene therapy will significantly impact the care that haemophilia patients require [29]. Initially, the decision arises of whether gene therapy is right for each individual patient. The MDT must ensure the patient is at the centre of the shared decision-making process and that education and counselling are available to each individual [29, 30]. In addition, the practicalities of administering gene therapy, including pre- and post-treatment assessments and product infusion, differ from current therapies [21].

Gene therapy necessitates multidisciplinary care, incorporating key roles for treating physicians, nurses, physiotherapists and psychologists, and additional specialities such as hepatologists [30, 31]. As such, an integrated approach is advocated, ensuring the required level of care is delivered efficiently at each stage of the patient journey [29]. The hub-and-spoke model may facilitate this when a patient lives at a distance from a haemophilia treatment centre (HTC) that is experienced in gene therapy. In this model, the hub is an HTC that prescribes and manages gene therapy and the spoke is an HTC without gene therapy experience, where pre- and post-infusion monitoring is performed; the model requires close communication between the two and sets a new precedent in the standard of haemophilia care [21, 30]. The haemophilia community within their national member organisations and societies, pharmaceutical industry and payers are also key partners throughout the process, providing further support via delivery of shared decision-making tools (e.g., The World Federation of Hemophilia Shared Decision-Making Tool [32]) and educational materials [29].

Herein, the authors present their opinions around the key concepts presented during the symposium at EHA 2022.

### Why is a 360-degree perspective important in gene therapy patient care?

#### Physician (Wolfgang Miesbach writes…)

With the introduction of novel treatment options, multidisciplinary care has become even more crucial. Gene therapy requires the involvement of specialities who may not have been routinely involved in haemophilia care and responsibilities and tasks may need redefining.

During the pre-administration period, relevant stakeholders should be involved in determining patient eligibility for treatment. For example, liver health status should be assessed by a hepatologist [21, 31]. Following administration, increased levels of inflammatory markers of the liver (alanine aminotransferase [ALT] elevation) require close monitoring and may necessitate corticosteroid administration to preserve transgene expression [31]. Corticosteroid therapy involves careful management to minimise the risk of adverse events [31], including tapering of doses to avoid secondary adrenal insufficiency from long-term use [33]. Furthermore, patients with pre-existing anti-AAV NAbs or those experiencing cytotoxic T-cell responses following treatment may require advice from an immunologist.

Prior to gene therapy treatment, psychologists play a vital role in exploring patient motivation for receiving gene therapy [30]. Furthermore, they can support patients who may be ineligible for gene therapy, have a lack of response or experience a loss of identity following an improvement in their disease.

As gene therapy may not only affect the patient themselves, the decision-making process should also involve caregivers and/or families. Family members may be best placed to provide a true picture of the patient’s current situation, and discussions should reflect on the existing treatment landscape and how the current approach to gene therapy would fit into the patient’s lifestyle.

#### Nurse (Greta Mulders writes…)

The haemophilia nurse has a central role in providing guidance and follow-up care, not only to those receiving gene therapy, but to all patients with haemophilia [34, 35]. As well as their clinical care skills, haemophilia nurses have specialist clinical knowledge and can provide support to patients and their families in a collaborative manner [34]. As nurses often have the first contact with patients, a nurse-patient relationship based on trust can be developed, with support/follow-up tailored to individual needs [34].

As the treatment landscape evolves, the provision of patient care is also transforming [29]. Healthcare providers often adapt to new strategies, technologies and procedures quickly, yet patients may not be as resilient in handling these changes, resulting in missed opportunities for patients to obtain a holistic view of their needs. A 360-degree perspective of the team members involved in patient care is key, enabling the MDT to have a full understanding of the patient journey. This allows each member of the MDT to engage the patient with the right message at each stage of their healthcare journey, using the most appropriate channels and suitable level of detail for the patient. One of the simplest ways healthcare professionals (HCPs) can increase patient engagement is to tailor the way they communicate to suit each individual patient. Published research has demonstrated that increased patient engagement leads to improved outcomes [36]. In some centres, patients may be included in multidisciplinary meetings; a UK Government response on the ‘Liberating the NHS: No decision about me, without me’ consultation provided various suggestions to increase patient involvement in the care process [37]. Effective involvement of the patient requires good communication with their healthcare professionals and may increase treatment compliance.

#### Patient (Daan Breederveld writes…)

**Fig. 1:**
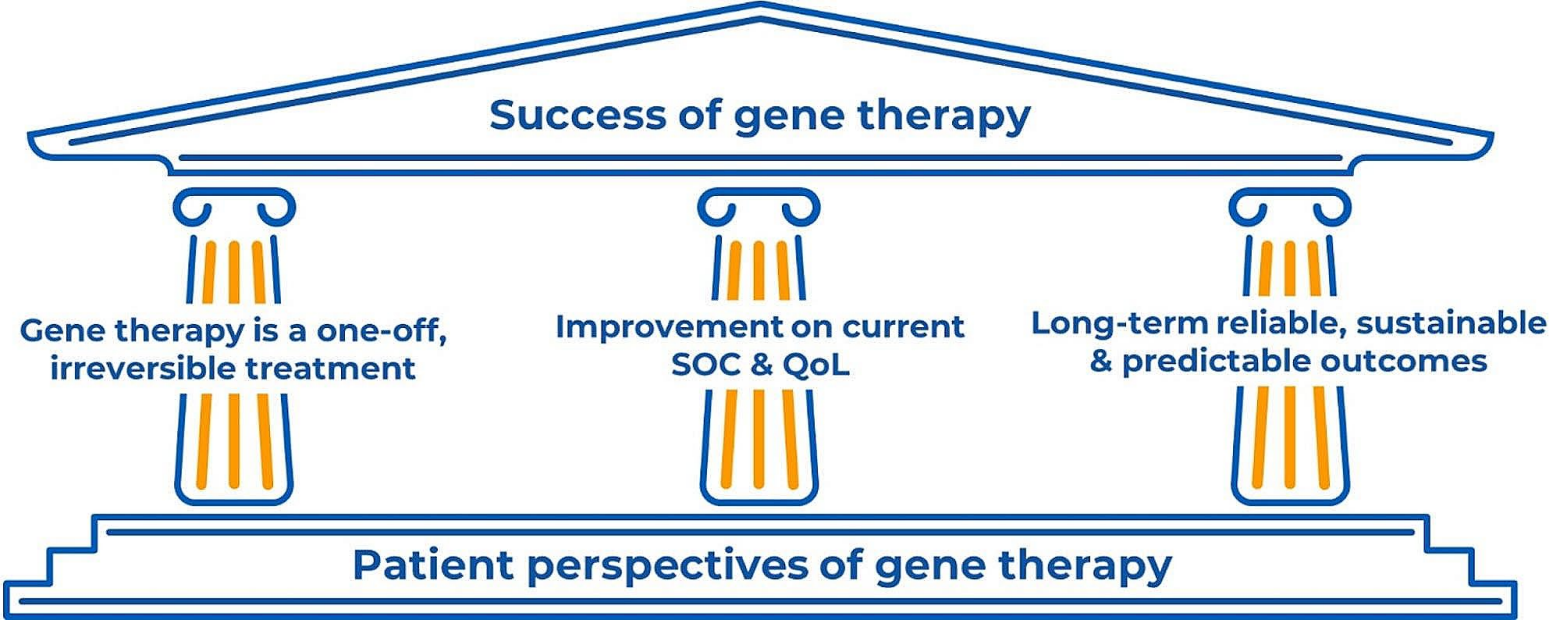
Why is the patient perspective on gene therapy important? From the perspective of the patient, gene therapy is a one-off, irreversible treatment that requires careful consideration. Factors in the success of gene therapy include improvement on the current standard of care and quality of life, as well as long-term reliable, sustainable, and predictable outcomes. QoL, quality of life; SOC, standard of care

In the current healthcare landscape, the patient perspective is of growing importance, especially for the success of gene therapy (Fig. 1). With the increasing use of various types of media due to global technological advancements, information is now available to a wider proportion of the population than ever before; however, not all sources are necessarily reliable. In addition, patient attitudes towards HCPs are increasingly critical; general acceptance of any medical treatment by patients is dependent on a well-informed team of HCPs with excellent communication skills.

A patient’s decision of whether to undergo gene therapy may be influenced by the current standard of care and disease burden, which varies significantly between countries and patients. Furthermore, psychological, cultural and/or religious aspects may influence a patient’s attitude towards gene therapy. As gene therapy is a one-off, irreversible treatment [20, 21], the decision-making process can take both time and effort from all involved. It is unlikely that patients will be able to manage this by themselves; I required support from my MDT, family, social and professional networks. The possibility that I would no longer be dependent on regular therapy to live was a major contributor to the decision-making process.

The challenge for gene therapy is to offer long-term, sustainable results [38]. Considering this and other uncertainties (e.g., short- and long-term side effects), patients must be provided with all pros and cons of treatment. This represents an unprecedented challenge in haemophilia care and emphasises the need for a new approach.

### From your perspective, what are your expectations for before/during/after gene therapy, and how should these be managed?

#### Physician (Wolfgang Miesbach writes…)

**Fig. 2:**
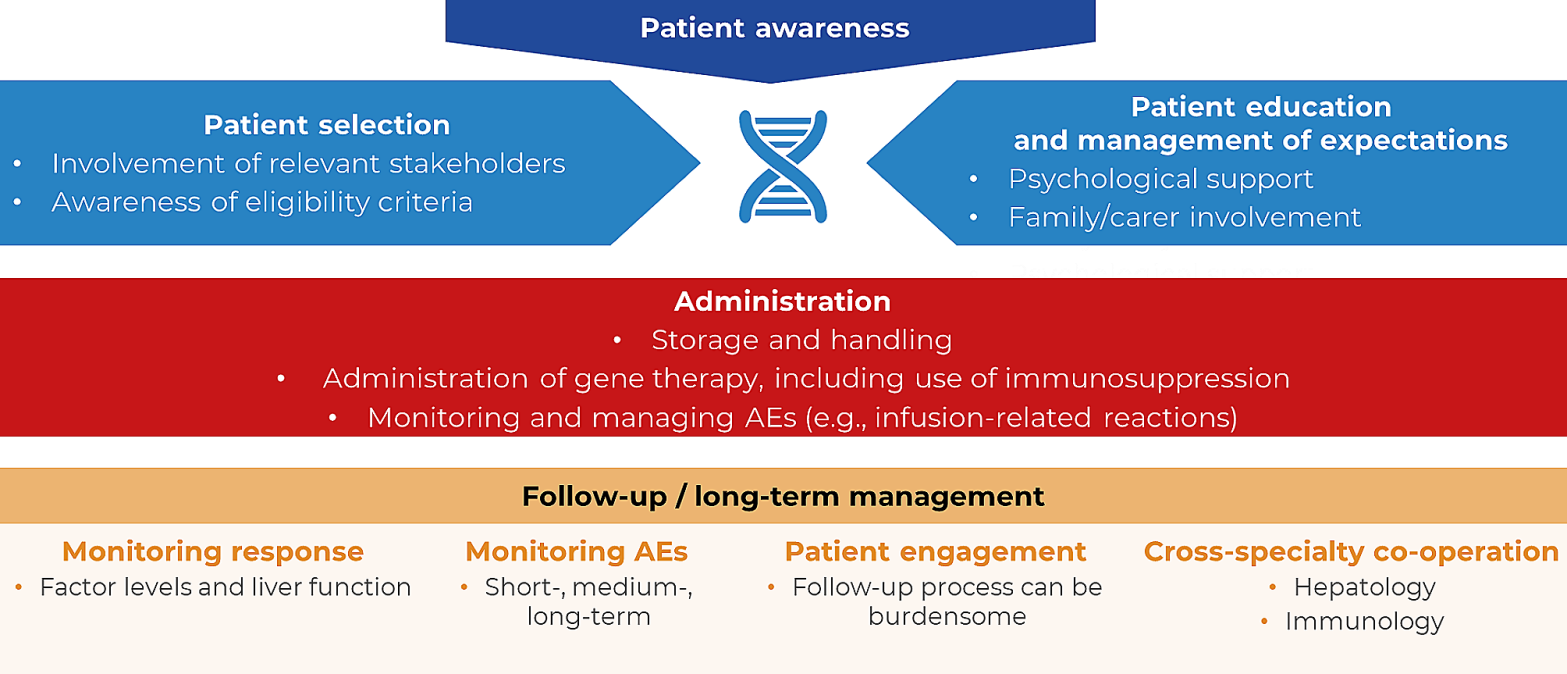
Challenges for haemophilia treatment centres. The challenges for haemophilia treatment centres involved in the management of gene therapy include patient awareness, administration logistics and follow-up/long-term patient management. AEs, adverse events

The major challenges surrounding gene therapy management for HTCs are summarised in Fig. 2. Firstly, patients and HCPs should be aware that any changes to the patient’s eligibility since the initial assessment could affect whether gene therapy can be administered. During administration, infusion-related reactions (IRRs), such as hypersensitivity reactions and fever, are possible [2, 6, 7]; patients should be informed about the risk of these occurring and physicians should have access to relevant guidelines. If IRRs occur, the balance of efficacy and safety must be considered. These reactions may be managed by prematurely stopping the infusion; however, this could result in inadequate treatment and may prevent the patient from receiving gene therapy in the future [2, 7, 20]. It is also important to consider that some effects may be related to anxiety surrounding the situation and not the gene therapy itself.

During the first year, a strict surveillance regimen focusing on factor levels and liver function is followed, with the most frequent monitoring during the first 3–6 months; less intensive long-term follow-up continues after the first year [10–13, 17]. Continuous assessment of the patient’s emotions and thoughts is also imperative [30]. To ensure realistic expectations, physicians should discuss these follow-up requirements with patients in advance of infusion day. This monitoring may simply replace the burden associated with their previous treatment. Home laboratory testing may reduce some of the inconvenience, although some HTCs may prefer for physicians to assess the patient each time. Electronic diaries can be a useful tool to aid collaboration between different centres, whilst providing transparency for the patient. It is essential that contact with patients is maintained following gene therapy, even if factor levels are normalised.

#### Nurse (Greta Mulders writes…)

Considering the possibility of providing long-term treatment, without the need for frequent infusions, gene therapy has the potential to incite a shift in the standard of care for haemophilia [38, 39]. However, favourable patient selection and discussions surrounding expectations are key for patients to gain the full benefit [38].

**Fig. 3:**
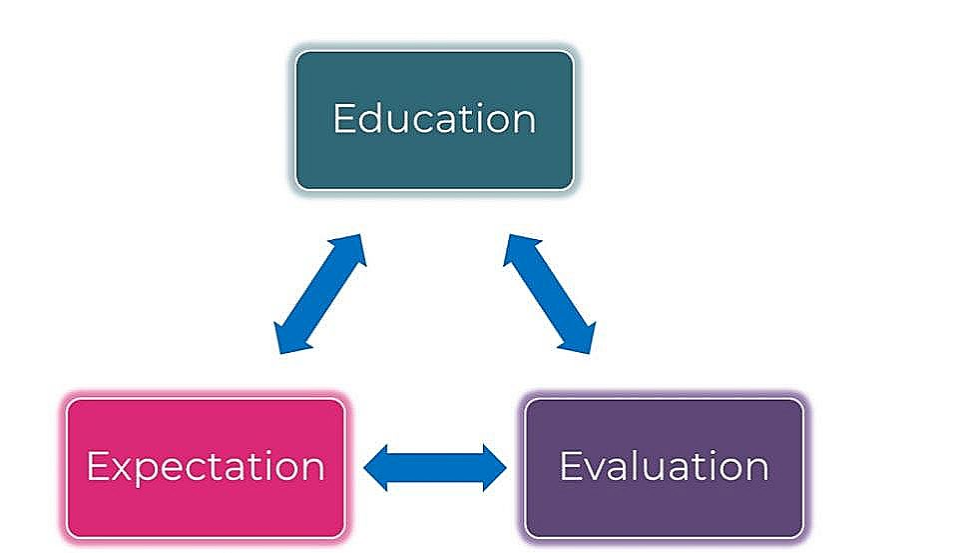
The ‘Triple E’ of the nurses’ role within the gene therapy process. The nurses’ role involves educating patients on gene therapy and exploring patient expectations, as well as evaluation of both components, allowing for further guidance and support where necessary. These three responsibilities are linked and should be continually undertaken throughout the whole gene therapy process

Nurses should fully support patients to form their own perspective of gene therapy; the ‘Triple E’ (education, expectation, and evaluation) of the nurses’ role in this process is shown in Fig. 3. To assist patients with their understanding of the gene therapy process, educational approaches and psychological therapy that continue throughout the whole patient journey should be implemented [40]. It should be noted that the long-term psychosocial effects of gene therapy require further research [40]. Additionally, expectations of patients and their family/carers must be continually explored from multiple angles, ensuring that they are realistic to avoid disappointment. The patients’ knowledge of gene therapy, their expectations and outcomes should be evaluated at various time points, allowing for further guidance and support where necessary. It is essential that these discussions cover all the factors that matter most to patients, including their preferences, needs and values. One UK study investigated the treatment characteristics that are important to people with haemophilia during the decision-making process, where treatment choice and effectiveness, safety, self-management, and quality of life were identified [41]. Previous research into patient-centred education for other diseases has demonstrated positive influences on outcomes, which may be a result of improved self-management [42–44].

#### Patient (Daan Breederveld writes…)

To manage expectations appropriately, each individual patient’s hopes and anticipations of gene therapy need to be known; HCPs should facilitate a dialogue in which their patients feel comfortable disclosing their thoughts. These discussions should include motivational aspects, guidance on accessing information, assessment of the quality of information studied and conversations surrounding all aspects of treatment [30]. HCPs need to understand each patient’s beliefs and perspectives, and explore the treatment outcomes that would be most important to them. Questions, such as “What do you think will happen following treatment?” and “How important is this to you?” should be used to form discussions. Early in the process, eligibility should be discussed to minimise disappointment in the scenario of a patient who is ineligible for their chosen gene therapy.

Additional conversations surrounding possible side effects and dealing with the unknown factors that influence outcomes should ensure a well-informed decision. Investigations into patient perspectives following gene therapy identified that side effects associated with immunosuppressive agents negatively affected patient satisfaction [40]. Other factors, such as lack of improvement in already damaged joints, may also contribute to disappointment with treatment. Influences on treatment satisfaction vary between patients, emphasising the importance of a thorough and open discussion around what outcomes each patient can/cannot expect. Throughout the decision-making process, opinions may vary; patients may dismiss thoughts following discussions with their MDT, but these may return at a later stage.

### What are the unknowns associated with gene therapy and how should these be managed?

#### Physician (Wolfgang Miesbach writes…)

From the physician’s perspective, the first unknown is being unable to predict individual patient factor levels following administration [20], which is a contrast to the relative ease of predicting factor levels when administering clotting factor concentrates [1]. The second unknown is how long factor expression will last; we await the outcomes of studies into long-term factor expression to be collected through Phase 4 post-marketing clinical studies and registries such as The World Federation of Hemophilia Gene Therapy Registry (WFH GTR; NCT04883710) [20, 45]. Furthermore, long-term safety is an uncertainty that patients should be made aware of, specifically the unknown risks of vector integration to the liver and potential development of malignancies [2, 20]. Long-term research will increase understanding of these unknowns, and thus, provide more transparency to future patients considering gene therapy.

#### Nurse (Greta Mulders writes…)

From the nurse’s perspective, the main concerns are the unpredictable changes in the patient’s physical and emotional health, as well as their lifestyle, that may occur due to gene therapy. Prior to treatment, patients may have worries regarding their suitability for gene therapy and, if ineligible, may question why and whether they will ever become eligible. In addition, they may have anxieties surrounding the efficacy of treatment and duration of factor levels [46]. It is not possible to predict whether patients will require immunosuppressive therapy in response to liver enzyme elevations [2, 20]. The potential psychological impact of steroid therapy should not be underestimated and is an area that most nurses should be familiar with [40]. The associated side effects should be discussed openly before initiating treatment; insomnia, weight gain and mood swings can have devastating effects for individuals and their families [40].

Transparent communication of uncertainties during the shared decision-making process is critical and should take into consideration patient education level, as well as cultural and time challenges [47]. An effective dialogue has the potential to further develop the trust between nurse and patient, allowing for an improvement in clinical outcomes [47].

#### Patient (Daan Breederveld writes…)

Clotting factor levels and duration of effect are some of the patient-reported unknown factors associated with gene therapy [41, 46]. The change in perceived illness and identity, and the possibility of side effects in the first few months following administration, are also uncertainties. In my experience, the worry of having too much clotting factor was another unknown concern.

In severe haemophilia, a minimal rise in factor levels will generally reduce bleeding frequency significantly and thus, perceived illness [48]. After reading the outcomes of other clinical studies and trying to predict what response I would have, I had hoped for a rise in clotting factor levels of up to 50% or more, but my actual increase was much less. I later recognised that no longer suffering with spontaneous bleeding was far more important to me than the rise in my factor levels. Also, my concern of being at risk of thrombosis due to supraphysiological clotting factor levels was reduced [39].

Concerns about the loss of identity is one of the reasons for patients not engaging with gene therapy, emphasising the importance of addressing this topic during decision-making discussions [46, 49]. Perceived changes in identity tend to play a role when higher standards of care are introduced. Patients may feel “normal” following gene therapy, in comparison to a lifetime of feeling unique, and will need to adapt to having a “haemophilia-free mind”. Living with haemophilia greatly impacts mindset and behaviour, resulting in identification with the former, chronic disease, even following gene therapy. People may take pride in living an “almost normal life” or undertaking “normal activities”, despite their disease or limitations. This state of mind is not unique for haemophilia [50]; “the disability paradox” is well-described in international literature [51]. One qualitative study reported that more than half of the participants interviewed described themselves as having an excellent or good quality of life, despite their moderate to severe disabilities [51].

As gene therapy is introduced, patients must be aware that unknown factors with no clear outlook may remain. Dealing with uncertainty is part of everyday life and individuals approach it differently. For some patients, altruism played a role in their decision to receive gene therapy during the clinical trials, despite the unknown effects [40]. As more gene therapies are approved for use, with many unknowns still surrounding them, these attitudes may change.

### What practical steps are needed before the roll out of gene therapy?

#### Physician (Wolfgang Miesbach writes…)

One of the most important practical steps is the collaboration between all involved HCPs (e.g., physicians, pharmacists and hepatologists), and between the different HTCs [30]. Following treatment, it should be clearly defined who and which centre is responsible for follow-up and management [21]. For example, for surveillance of ALT elevations and for establishing a protocol for immunosuppressive therapy implementation. At the regional level, a certification process should be put in place for centres, based on whether they administer gene therapy and/or care for patients after gene therapy administration [21]. Hub centres should partner with patient organisations to address open questions regarding gene therapy and to offer support to patients [21, 52].

Finally, any long-term effects must be recorded using standardised data registries (e.g., WFH GTR) [29, 45], and a procedure should be established for the management of vector integration and development of malignancy, if this situation arises.

#### Nurse (Greta Mulders writes…)

The close nurse-patient relationship remains the same when gene therapy is the choice of treatment. However, nurses may experience new challenges such as coordinating the patient journey and ensuring that Hub and Spoke centres share all necessary information [30]. In clinical trials, recurring meetings were organised to discuss all patients who were undergoing gene therapy; these discussions consisted of laboratory outcomes, clinical setting, the patient’s mental state and any other particularities. This is one example of how the MDT could approach effective collaboration.

Prior to the roll out of gene therapy, standard operating procedures covering the whole gene therapy process should be established, including the requirement for education/training of staff to ensure a consistent level of knowledge [29]. Education could be provided as written materials, e-learnings, webinars or podcasts from reliable sources (e.g., from the European Association for Haemophilia and Allied Disorders [EAHAD] [53]). Additionally, policies should detail the organisation of hospital admissions and the management of interactions with other departments (e.g., pharmacy and laboratory). Guidance on handling the gene therapy product should also be provided. On infusion day, a dedicated nurse should be available at all times for support and guidance [30], and the physician should be present during the infusion [54].

A procedure defining laboratory testing requirements prior to and following treatment should be developed; for example, the spoke centre must have continuous laboratory testing available for factor level monitoring. For each individual gene therapy, anti-AAV NAbs should be measured using the same laboratory test in all centres to allow for standardisation and specificities to the prescribed product. Effective communication between centres will also aid planning of follow-up visits, whilst ensuring feasibility for the patient. The post-infusion period consisting of life changes and follow-up monitoring requires careful attention. Patients will have various questions throughout the process; following gene therapy, these may focus on safety, factor levels and registry requirements [55]. To enhance communication and alleviate patients’ worries, opportunities for patients to ask questions to their MDT should be offered; mobile applications are one method of facilitating this prompt interaction.

#### Patient (Daan Breederveld writes…)

Most importantly, all eligible patients should have access to gene therapy along with reliable information to facilitate decision-making [21, 30]. HCPs should be well-trained in effective communication, and should be adequately informed of the latest clinical trial data as well as the possible side effects of therapy. The gene therapy process should be carefully planned and thoroughly understood by all involved. Patients should be aware of the logistical elements, which may involve lengthy follow-up consultations, to enable them to factor this into their private and professional lives.

## Conclusion

This review highlights the key considerations required to engage with haemophilia gene therapy effectively, from a 360-degree perspective of the physician, nurse and patient. It is essential for both patients and providers to be familiar with the perspectives of other members of the MDT prior to their involvement with the gene therapy process. Although the impact on quality of life seems promising, patients face a complex decision-making process. Different challenges are presented to patients when considering these new therapies, such as whether gene therapy is right for them at their current stage of life considering the follow-up commitments and unknown factors. All authors considered that the key to success for gene therapy includes collaboration and tailored education across the MDT (including patients and their carers/families), starting early in the process and continuing throughout the long-term follow-up period. Additionally, patient expectations, which may surround eligibility, follow-up requirements and treatment outcomes, should be continually explored. During these ongoing discussions, transparent communication of the unknown factors, such as anticipated clotting factor levels, long-term factor expression and safety, post-administration follow-up which may last decades, and psychological changes, is critical. To ensure efficiency and comprehensiveness, clearly‑defined protocols should outline the whole process, which should include the recording and management of long-term effects. The future after approval of haemophilia gene therapies remains to be seen; we await real-world evidence for key aspects such as pre-existing anti-AAV NAb testing, challenges for HTCs, requirements for the use of registries and alternative payment options.

.

## Data Availability

Not applicable.

## Abbreviations

AAV: Adeno-associated virus
ALT: Alanine aminotransferase
EAHAD: European Association for Haemophilia and Allied Disorders
EC: European Commission
EHA: European Hematology Association
FDA: US Food and Drug Administration
FIX: Factor IX
FVIII: Factor FVIII
HCPs: Healthcare professionals
HTC: Haemophilia treatment centre
IRRs: Infusion-related reactions
MDT: Multidisciplinary team
MHRA: UK Medicines and Healthcare products Regulatory Agency
NAbs: Neutralising antibodies
WFH GTR: The World Federation of Hemophilia Gene Therapy Registry
